# Evaluating the efficacy of biological and conventional insecticides with the new ‘MCD bottle’ bioassay

**DOI:** 10.1186/1475-2875-13-499

**Published:** 2014-12-16

**Authors:** Eleanore D Sternberg, Jessica L Waite, Matthew B Thomas

**Affiliations:** Center for Infectious Disease Dynamics and Department of Entomology, Pennsylvania State University, University Park, PA USA

**Keywords:** Vector control, Olyset, Bendiocarb, Chlorfenapyr, Fungal entomopathogen, Resistance, WHOPES

## Abstract

**Background:**

Control of mosquitoes requires the ability to evaluate new insecticides and to monitor resistance to existing insecticides. Monitoring tools should be flexible and low cost so that they can be deployed in remote, resource poor areas. Ideally, a bioassay should be able to simulate transient contact between mosquitoes and insecticides, and it should allow for excito-repellency and avoidance behaviour in mosquitoes. Presented here is a new bioassay, which has been designed to meet these criteria. This bioassay was developed as part of the Mosquito Contamination Device (MCD) project and, therefore, is referred to as the MCD bottle bioassay.

**Methods:**

Presented here are two experiments that serve as a proof-of-concept for the MCD bottle bioassay. The experiments used four insecticide products, ranging from fast-acting, permethrin-treated, long-lasting insecticide nets (LLINs) that are already widely used for malaria vector control, to the slower acting entomopathogenic fungus, *Beauveria bassiana*, that is currently being evaluated as a prospective biological insecticide. The first experiment used the MCD bottle to test the effect of four different insecticides on *Anopheles stephensi* with a range of exposure times (1 minute, 3 minutes, 1 hour). The second experiment is a direct comparison of the MCD bottle and World Health Organization (WHO) cone bioassay that tests a subset of the insecticides (a piece of LLIN and a piece of netting coated with *B. bassiana* spores) and a further reduced exposure time (5 seconds) against both *An. stephensi* and *Anopheles gambiae*. Immediate knockdown and mortality after 24 hours were assessed using logistic regression and daily survival was assessed using Cox proportional hazards models.

**Results:**

Across both experiments, fungus performed much more consistently than the chemical insecticides but measuring the effect of fungus required monitoring of mosquito mortality over several days to a week. Qualitatively, the MCD bottle and WHO cone performed comparably, although knockdown and 24 hour mortality tended to be higher in some, but not all, groups of mosquitoes exposed using the WHO cone.

**Conclusion:**

The MCD bottle is feasible as a flexible, low-cost method for testing insecticidal materials. It is promising as a tool for testing transient contact and for capturing the effects of mosquito behavioural responses to insecticides.

## Background

Control of adult mosquito vectors is central to the efforts to eradicate malaria [1]. The ability to conduct reliable laboratory tests of insecticides (chemical or biological) against adult mosquitoes is a key step in product development and ongoing insecticide resistance monitoring. The standard tools to test insecticides, insecticide treated materials, and monitor resistance are the World Health Organization (WHO) tube, the WHO cone, the Centers for Disease Control (CDC) bottle assay [2] and, to a lesser extent, the WHO wireball assay and tunnel test [3–5]. These methods are used for different kinds of tests, and all of them have a different set of benefits and drawbacks. For example, the WHO tube and the CDC bottle assays are both used for detecting and characterizing insecticide resistance in mosquitoes using a controlled dose of insecticides, either applied to paper (WHO tube) or coated on the inside of the bottle (CDC bottle). The two methods differ in that the WHO tube relies on equipment that must be acquired from a single source, while the CDC assay uses glass bottles that are readily available as laboratory equipment and can be prepared on site by the end user. There is a benefit that comes with using inexpensive, locally available materials e.g. [6, 7], rather than having to source WHO materials, and consequently the CDC bottle lends itself to greater flexibility but this flexibility comes with a potential cost to quality assurance and control when compared to the WHO tubes and standardized insecticide-impregnated papers. Unlike the WHO tube and the CDC bottle assay, the WHO cone can be applied to a wide variety of surfaces, including insecticide treated bed nets (ITNs) and house walls that have been treated by indoor residual spraying (IRS). Because it can be used on these kinds of treated materials, the WHO cone can be used to test the effect of insecticides formulated in a way that mosquitoes would actually encounter in the field. The WHO wireball assay and tunnel test can also be used to evaluate ITNs, but the two methods differ in an important way: the wireball assay is a contact bioassay like the cone test, where mosquitoes are held in contact with the material, while the tunnel test incorporates a behavioural component by releasing a mosquito in a tunnel with a live host behind a piece of experimentally damaged netting. The mosquito can choose to avoid the netting and forgo the possibility of a blood meal, or attempt to navigate through the holes and gain access to the host. Despite their differences, it is worth noting that all of these tools use a protocol that calls for a relatively long exposure time (depending on the test, 3 minutes to 1 hour exposures or up to 12–15 hours in the case of the tunnel test) and relatively quick read outs – knockdown measured 1 hour post exposure and mortality measured 24 hours post exposure.

**Figure 1:**
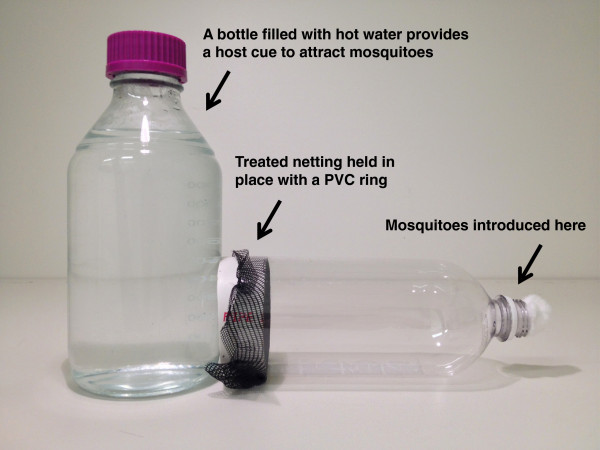
**The MCD bottle bioassay.** A clean 1-L plastic bottle with the bottom cut off and a piece of netting held in place with a ring of PVC tube. The netting can be a piece cut from an LLIN, netting dipped in liquid formulations of insecticides, or electrostatically charged netting coated with dry formulations of insecticides, including fungal spores. Mosquitoes are introduced via an aspirator at the bottle opening (right), which can then be sealed using a clean piece of cotton. Mosquitoes are then allowed to recruit to a heat or odour cue placed behind the netting (left), for example a glass jar filled with hot water.

Presented here is an alternative method for evaluating treated materials like netting. The assay, referred to as the MCD bottle bioassay, consists of a clear plastic water or soda bottle with the bottom cut off (Figure 1). A ring of PVC tube fits into the open bottom of the bottle and holds in place a piece of netting or treated material. This ring could also be substituted with a rubber band around the outside of the bottom of the bottle to hold material in place. Mosquitoes are introduced into the neck of the bottle (individually or in groups) using an aspirator, and a source of heat or odour is placed next to the bottom end of the plastic bottle to attract the mosquitoes to the treated substrate.

This bioassay was developed as part of the Mosquito Contamination Device (MCD) project [8], which aims to develop novel, low-cost technology for controlling malaria vectors in resource-poor settings. Development of this bioassay was partly motivated by the need for a technique that could be used to simulate aspects of a more natural interaction between mosquitoes and insecticides. These aspects include recruitment to netting in response to attractive stimuli (similar to the tunnel test), excito-repellency and avoidance behaviour, and transient contact between mosquitoes and treated materials. There is scant information describing exactly how mosquitoes interact with different interventions in the field but in principle, a mosquito might alight on treated surfaces for only seconds when exploring the surface of a bed net, or when attempting entry through a barrier such as window screen or treated curtain. Moreover, mosquitoes exhibit excito-repellency and avoidance behaviours in the presence of certain insecticides and, upon knockdown, might be as likely to fall away from a treated substrate as onto it. The MCD bottle has several characteristics that make it useful for simulating these kinds of interactions. The transparency of the plastic bottle allows for close observation of behaviour during mosquito recruitment and contact with the net. Mosquitoes can be introduced for a set time, with the actual duration of exposure dependent on behaviour, or mosquito behaviour can be monitored and the mosquitoes removed by aspirator when a certain cumulative contact time with the substrate has been reached. Moreover, with traditional WHO cone and tube assays, mosquitoes remain in contact with the substrate if they are knocked down. The MCD bottle has the advantage that, when mosquitoes are knocked down, they will no longer be in contact with the active, as might be expected for a mosquito in many field settings.

Another motivating factor for developing this bioassay was the need for reduced cost and improved accessibility. Similar to the CDC bottle bioassay, the MCD bottle protocol can be carried out using locally available materials – and in fact, uses materials that are less expensive and easier to obtain than glassware – and it can be adapted to test a wide variety of insecticides. Additionally, because the materials used in the MCD bottle bioassay are inexpensive and easy to acquire, bottles can be discarded after use, thereby reducing the risk of contamination that comes with re-use of WHO tubes and cones.

Presented here is a set of proof-of-concept experiments using chemical and biological actives with the MCD bottle bioassay. The aim is partly to demonstrate the utility of the assay technique and, more fundamentally, to explore the relative efficacy of diverse actives under different exposure scenarios and assess the appropriateness of typical endpoints (i.e., 1 hour knockdown and 24 hours mortality) for actives with different modes of action.

## Methods

### Mosquitoes and active ingredients

*Anopheles gambiae* (Keele strain [9], acquired from a colony maintained at Johns Hopkins University, Maryland, USA) and *An. stephensi* (acquired from a colony maintained at NIH, Bethesda, Maryland, USA) mosquitoes used in these experiments were reared under standard insectary conditions at 27°C and 80% relative humidity with a 12 L:12D photoperiod cycle. Larvae were fed ground TetraFin flakes and adults were fed a sugar water solution consisting of 10% glucose supplemented with 0.05% para-aminobenzoic acid (PABA). Adult mosquitoes two to five days post emergence were used in all experiments.

The list of chemical and biological actives and formulations used in the different experiments, together with relevant controls, are given in Table 1. Netting was dipped in either 0.1% bendiocarb (a carbamate) or 1% chlorfenapyr (a halogenated pyrrole), coated with a fungal entomopathogen previously shown to have potential as a biological alternative to chemical active ingredients [10–14], or netting samples were cut from a long-lasting, insecticide-impregnated net (LLIN). The bendiocarb and chlorfenapyr were formulated in a mix of oils and acetone according to WHO protocols [3] and applied to nylon netting by dipping. Netting was submerged in the formulations for two minutes and then removed and left to dry for 24 hours prior to testing. Fungal exposures were done with dry spores of *Beauveria bassiana* (GHA strain passaged through *Anopheles* mosquitoes) produced at the Pennsylvania State University (see [14] for production methods). Spores were applied to electrostatically charged polyester netting (180 holes/in^2^ with a modified Pollentex^®^ coating, acquired from In2Care^®^, Wageningen, The Netherlands) by shaking netting with an excess of spores together in a plastic bag. The electrostatic charge causes the spores to attach to the netting but to remain accessible to insects on contact. At saturation (i.e., when the netting cannot take up further spores), the netting holds approximately 5.7 g spores per sq m. The LLIN was either Olyset or Pramex, both of which are produced using the same technology and have 2% permethrin incorporated into polyethylene net fibres.

**Table 1:** **Netting with insecticides tested against** ***Anopheles stephensi*** **using the MCD bottle bioassay**

Netting type	Active	Formulation
Electrostatically charged polyester	None	Control
Nylon	None	Olive oil + acetone control
Electrostatically charged polyester	*Beauveria bassiana*	Powder
Nylon	0.1% bendiocarb	Olive oil + acetone
Nylon	1% chlorfenapyr	Silicone oil + acetone
Polyethylene	2% permethrin	Olyset or Pramex impregnated LLIN

### Comparison of actives and exposure times

The first set of experiments examined how exposure time influenced the efficacy of a range of active ingredients. Mosquitoes (*An. stephensi*) were exposed to one of the six treatments listed in Table 1 using the MCD bottle bioassay (Figure 1) made from a 1 L bottle with an exposure area of 50.3 cm^2^. A glass jar filled with hot tap water (approximately 35°C) was used as an attractant. Preliminary testing suggested that even a small difference between the water and ambient temperature, on the order of a few degrees, was sufficient to cause recruitment but the water was still replaced frequently throughout testing to maintain water temperature as much as possible. Mosquitoes were aspirated into the bottle in groups of ten using dedicated aspirators for each treatment group to prevent possible cross-contamination. Exposure times were one, three or 60 minutes; three and 60 minutes are recommended within WHO protocols, for testing residual activity on netting with WHO cones and when assessing diagnostic concentrations using WHO tubes respectively, whereas one minute represents a more transient exposure. There were six replicates of ten mosquitoes for each combination of treatment group and exposure time, for a total of 480 mosquitoes. Exposure was based on time within the bottle, not necessarily the exact time mosquitoes were in contact with the netting. After exposure, mosquitoes were held in paper cups and provided with cotton balls soaked with sugar water, replaced daily. Knockdown was recorded one hour after exposure and cups were checked daily for seven days post exposure to measure daily survival. Mortality was monitored up to one week post exposure.

### Comparing the outcome of exposures with the WHO cone and the MCD bottle

A second follow-up experiment was carried out to directly compare the WHO cone test and the MCD bottle assay. This experiment compared netting coated with fungal spores, a piece of an LLIN, and a piece of clean netting as a control. Both *An. gambiae* and *An. stephensi* were exposed to these materials with a range of exposure times; three minutes as recommended by the WHO protocol [3], as well as the short exposure times of one minute used in the first experiment and an even further reduced contact time of five seconds. Exposure period was based on time within the bottle, not the exact time mosquitoes were in contact with the netting. Exposures were done in ten groups of five mosquitoes, again per the WHO guidelines, for a total sample size of 1,800 mosquitoes. After exposure, mosquitoes were held in paper cups and provided with cotton balls soaked with sugar water, replaced daily. Cups were checked one hour after exposure to measure knockdown, and then checked daily to measure mortality. For this experiment, mortality monitoring was extended to four weeks post-exposure.

### Statistical analysis

All statistical analyses were carried out using R v. 3.0.1. For the comparison of actives and exposure times using the MCD bottle bioassay, generalized linear models (GLM) were used with a quasibinomial error distribution to correct for overdispersion in the data. The model included the type of insecticide, the exposure time and days post-exposure (day 0, day 1 and day 7) as explanatory variables and survival (yes/no) as the outcome. The three time points correspond to the standard WHO time points for knockdown one hour post-exposure (day 0) and mortality at 24 hours post-exposure (day 1), while the time point at day 7 was included to illustrate the differences between chemical insecticides and fungus. To account for any block effects, exposure day (1 through 4) was also included, as well as all two-way interactions between exposure date and the three explanatory variables. Additionally, interactions between insecticide and exposure time, and insecticide and days post exposure were included. If interaction terms were not significant (p >0.05), they were removed from the final model.

For the comparison of the WHO cone bioassay and the MCD bottle bioassay, GLMs with quasibinomial error structures were again used to test the effect of different actives and difference exposure method (WHO cone *versus* MCD bottle bioassay) on knockdown 1 hour post-exposure, and mortality 24 hours post-exposure. In both cases, models included insecticide (LLIN or fungus), exposure time (5 seconds, 1 minute, 3 minutes), and exposure method (cone or bottle). The interaction between insecticide and exposure method, and insecticide and exposure time were included, considering exposure day and all possible two-way interactions with exposure day to account for block effects.

For both experiments, the ‘survival’ package in R was used to create Kaplan-Meier survivorship curves and to fit non-parametric models with Cox Proportional Hazards distributions to assess differences in survivorship.

## Results

### Comparison of actives and exposure times

**Figure 2:**
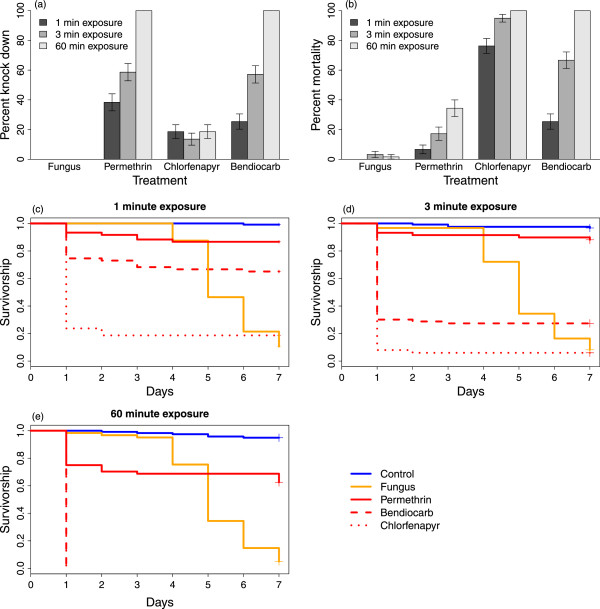
**Comparison of insecticidal active ingredients using the MCD bottle bioassay. (a)** Knockdown 1 hour post exposure, **(b)** mortality 24 hours post exposure, and **(c-e)** survival curves for *An. stephensi* exposed to actives for 1, 3 or 60 minutes, using the MCD bottle assay. Bars in **(a)** and **(b)** represent per cent knockdown or mortality ± SE and lines in **(c-e)** are Kaplan-Meier survivorship curves.

Overall, mortality was low in the control group (0% mortality on day 0 and 4.6% on day 7, averaged across the three exposure times). The two control groups did not differ and therefore were combined as a single group for subsequent analyses. For mosquitoes exposed to an active, the type of active (fungal or chemical) had a significant effect on knockdown and mortality over seven days (Figure 2a and b; F_3,196_ = 30.6, p <0.001) and there was a significant interaction between the type of active and the time since exposure (active x time interaction: F_6,196_ = 44.3, p < 0.001), demonstrating the difference between a fast-acting chemical insecticide and a slower acting biological insecticide like fungus. One day after being exposed to chemical insecticides, survival in the fungus group was comparable to the control group (2% mortality in the fungus group on day 0). However, by day 7, mortality in the fungus was as high, or higher, than the mortality in mosquitoes exposed to chemical insecticides (93.3% mortality on day 7 with fungus; mortality on day 7 ranging from 21.6% to 93.5% with different chemical insecticides). Moreover, the impact of chemical insecticides on mosquito survival was limited to the first 24-hour period following exposure, indicating that if a mosquito survived at least one day after exposure it was no more likely to die than a mosquito in the control group (Figure 2c). Even among the three chemical insecticides, there were noteworthy differences in their effects over time. Permethrin initially knocked down up to 100% of the mosquitoes that were exposed, but by the following day, mortality was less than 40%. In contrast, chlorfenapyr initially had a relatively minor impact on mosquito knockdown compared to permethrin and bendiocarb, but by 24 hours, mortality in mosquitoes exposed to chlorfenapyr was higher on average than mosquitoes exposed to either permethrin or bendiocarb. Unlike either permethrin or chlorfenapyr, there were no differences in knockdown at one hour or mortality at 24 hours in mosquitoes exposed to bendiocarb (Figure 2a and b).

Exposure time (1 minute, 3 minutes and 1 hour) also had a significant effect on mortality (F_2,196_ = 25.27, p < 0.001) and the interaction between exposure time and insecticidal compound was significant (F_6,196_ = 9.70, p < 0.001). One possible explanation for this interaction is that the effect of an insecticidal particle, like fungal spores, is less dependent on contact time. This might be because particles are transferred even with the brief contact, while insecticides impregnated into net fibres only act when there is direct contact with mosquitoes and consequently are more dependent on exposure time. If this is the case, insecticidal powders, either biological or chemical, might be a good option where there is only transient contact. For this experiment, there was no significant effect of exposure day (block), or any significant interactions between exposure day and insecticide, exposure time, or days post-exposure.

When daily survival was examined using Cox proportional hazard models, the results were consistent with the analysis of mortality at three time points. Survival in mosquitoes exposed to chemical insecticides or fungus was lower than control mosquitoes (Figure 2c), with the higher hazard ratio (HR) in the group exposed to chlorfenapyr (HR = 164, 95% Confidence interval, CI = (88.5, 307.4), p <0.001) and the lowest in the group exposed to the permethrin-treated LLIN (HR = 8.21, CI = (4.20, 16.0), p <0.001). Not surprisingly, survival was also significantly affected by exposure time, with the highest hazard ratios in mosquitoes exposed for 60 minutes (HR = 3.38, CI = (2.67, 4.28), p <0.001).

### Comparing WHO cone and MCD bottle in rapid exposure bioassays

**Figure 3:**
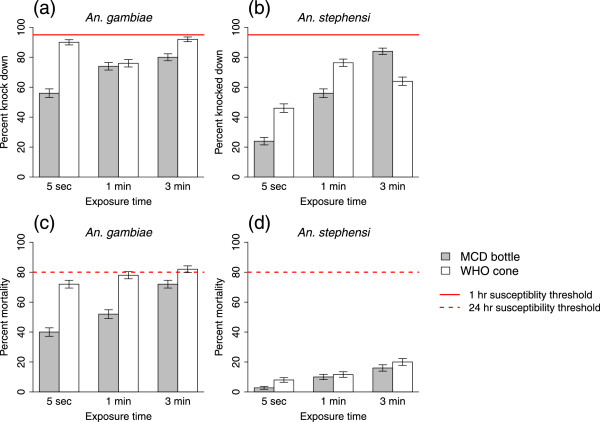
**Immediate effects of exposure to a long-lasting insecticidal net using the MCD bottle bioassay and WHO cone.** Per cent knockdown ± SE **(a and b)** and per cent mortality ± SE at 24 hours **(c and d)** for *An. gambiae* and *An. stephensi* exposed for 5 seconds, 1 minute or 3 minutes to a piece of LLIN.

**Figure 4:**
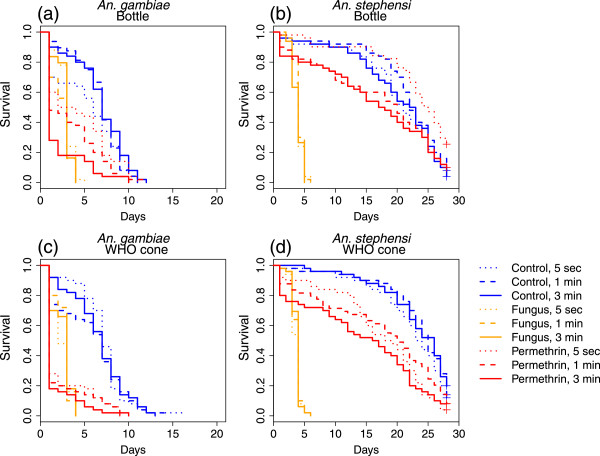
**Longer term effects of exposure to a long-lasting insecticidal net or fungus using the MCD bottle bioassay and WHO cone.** Kaplan-Meier survivorship curves for *An. gambiae* **(a and c)** and *An. stephensi* **(b and d)** exposed to a piece of LLIN or a piece of netting coated with fungus for 5 seconds, 1 minute or 3 minutes.

The results of the comparison between the MCD bottle and the WHO cone assay for the LLIN exposures are shown in Figures 3 and 4. Regardless of the exposure method, exposure time, or species (*An. stephensi* or *An. gambiae*), no group met the 95% knock down threshold for LLIN efficacy established by the WHO. Likewise, of the mosquitoes exposed to the LLIN for three minutes, per WHO protocol, only *An. gambiae* exposed using the WHO cone exceeded the 80% mortality threshold; however, the average mortality in this treatment group was still only 82%. This suggests that, in certain cases, the MCD bottle bioassay might produce a more conservative estimate than the WHO cone although this was not necessarily true across all groups.

Quantitatively, the exposure method did significantly affect the per cent knockdown in *An. gambiae* (combined across exposure times there was 70% knockdown in mosquitoes exposed with a MCD bottle compared to 86% knockdown with a WHO cone; F_1,53_ = 12.3, p = 0.001), but not for *An. stephensi* (55% knockdown in mosquitoes exposed with a MCD bottle compared to 61% knockdown with a WHO cone; F_1,52_ = 1.32, p = 0.255). Similarly, exposure method significantly affected per cent mortality after 24 hours in *An. gambiae* (55% mortality with bottle exposures compared to 77% mortality with cone exposures; F_1,52_ = 17.7, p <0.001) but not in *An. stephensi* (9% mortality with bottle exposures compared to 13% mortality with cone exposures; F_1,50_ = 1.60, p = 0.212). The difference between the WHO cone and MCD bottle was particularly pronounced for the short (5-second) exposure group, where there was a >20% increase in mortality in the group of *An. gambiae* exposed with the cone compared to those exposed with the bottle. A possible explanation for this observation is that the volume of the WHO cone is smaller than that of the MCD bottle, and transferring mosquitoes in and out of the cones increases accidental contact with the netting beyond the designated five seconds.

Overall, there was a significant effect of exposure time (5 seconds, 1 minute or 3 minutes) on both knockdown and mortality across both exposure methods and mosquito species (knockdown in *An. gambiae*: F_2,53_ = 3.18, p = 0.049 and in *An. stephensi*: F_2,52_ = 14.8, p <0.001; mortality in *An. gambiae*: F_2,52_ = 3.37, p = 0.042 and in *An. stephensi*: F_2,57_ = 3.83, p = 0.028). There was also a significant interaction between exposure method and exposure time for knockdown in both species (in *An. gambiae*: F_2,53_ = 3.87, p = 0.027 and in *An. stephensi*: F_2,57_ = 6.13, p = 0.004) but this interaction was not significant for mortality in either species. This is probably because per cent knockdown did increase with increasing exposure time in mosquitoes exposed using the MCD bottle but not necessarily in mosquitoes exposed using the WHO cone.

Similar to the results obtained from the first experiment, fungus was highly effective in killing mosquitoes overall, just over a longer time period than chemical insecticides. When Cox proportional hazard models were used to analyse survivorship, both exposure to LLIN and exposure to fungus had a significant effect on survivorship when compared to mosquitoes exposed to untreated netting (LLIN: HR = 2.06, CI = (1.50, 2.84), p <0.001; fungus: HR = 3.90, CI = (2.77, 5.49), p <0.001) but interestingly, in this analysis there was no significant difference between exposure using a WHO cone compared to an MCD bottle (HR = 0.890, CI = (0.707, 1.12), p = 0.324).

## Conclusion

These results demonstrate the feasibility of the MCD bottle assay as a novel method for testing insecticidal materials. The aim is not to propose this bioassay as a replacement for existing methods, such as the WHO cone bioassay, but given that it is easy to set up and requires no specialized equipment, it could provide a useful additional method. Moreover, features of the MCD bioassay make it a promising method for testing mosquito behavioural responses, for example in response to excito-repellent insecticides or attractants, and in situations where transient contact is of interest. Future experiments could include quantifying the time spent in contact with treated netting, to discover repellent effects of novel contaminants, and measuring the cumulative effects of repeated transient contact, compared with constant contact for an equivalent length of time. It would also be possible to evaluate realistic contact behaviours comparing mosquitoes with different resistance mechanisms. Little is known about how different levels and mechanisms of resistance affect host-searching behaviour and subsequent contact with LLINs (e.g. see [15]) or how different types of LLIN - including next generation products like the Olyset Plus net - might influence contact rates. The MCD bottle bioassay could easily be modified, for example by joining together multiple bottles to increase searching range or using larger bottles to increase volume, if it was necessary to allow for more diverse, less constrained behaviours.

Additionally, the experiments presented here demonstrate three general points relevant to all methods of testing actives. First, the time point at which the outcome of exposure is measured can change the conclusion for the efficacy of an active and, therefore, must be carefully considered when designing experiments and setting guidelines for testing. The WHO acknowledges this point for slower acting chemical insecticides like chlorfenapyr [4] and it is clear that that the testing time frame should be further extended for biological actives like fungus.

Second, these experiments show that exposure time had a significant effect on knockdown and mortality of mosquitoes exposed to LLINs, while exposure time appeared to be less important with fungus. The exact reasons for the relative insensitivity of fungal exposure are yet to be resolved, but it appears that the dose-transfer from the electrostatic netting is very efficient and almost any contact is sufficient to exceed the lethal dose. A possible analogy here is that mosquitoes pick up dry spores on their tarsi like grains of sand on a wet foot - as soon as the foot touches the sand it becomes coated and putting it back into the sand will not add any more grains. Previous research has also shown material treated with fungal spores to be attractive to adult mosquitoes [16], whereas pyrethroids tend to be excito-repellent. Whether efficient dose-transfer extends to other types of active ingredients, such as powder formulations of insecticides, is worth investigating.

Third, this study used longstanding laboratory strains of mosquitoes that have not been exposed to insecticides for many generations and thus the expectation was that these strains would be wholly susceptible to insecticides. Contrary to this expectation, both colonies, and the *An. stephensi* colony in particular, exhibited signs of resistance and would be considered resistant against Olyset nets by current standards using the WHO cone test. The WHO standard recognizes that testing with cones might not be sufficient to measure efficacy of bed nets, and suggests that tunnel tests are an alternative for measuring impact via effects on blood feeding inhibition [5]. It should be noted, however, that there are a number of prior studies that show very high levels of knockdown and mortality following exposure in a WHO cone to Olyset and other brands of LLINs e.g. [17–21]. Many of these trials were done using the Kisumu strain of *An. gambiae*, which is known to be highly susceptible. The observation that the strains used in this study were not fully susceptible raises the possibility that other laboratory strains that are considered nominally susceptible and maintained without selection may also exhibit some level of resistance to chemical insecticides when tested using a WHO cone.
